# Simple silicone chamber system for *in vitro* three-dimensional skeletal muscle tissue formation

**DOI:** 10.3389/fphys.2013.00349

**Published:** 2013-11-28

**Authors:** Celia Snyman, Kyle P. Goetsch, Kathryn H. Myburgh, Carola U. Niesler

**Affiliations:** ^1^Discipline of Biochemistry, School of Life Sciences, University of KwaZulu-NatalPietermaritzburg, South Africa; ^2^Department of Physiological Sciences, University of StellenboschStellenbosch, South Africa

**Keywords:** three-dimensional assays, tissue engineering, hydrogel constructs, *in vitro* skeletal muscle tissue

## Abstract

Bioengineering skeletal muscle often requires customized equipment and intricate casting techniques. One of the major hurdles when initially trying to establish *in vitro* tissue engineered muscle constructs is the lack of consistency across published methodology. Although this diversity allows for specialization according to specific research goals, lack of standardization hampers comparative efforts. Differences in cell type, number and density, variability in matrix and scaffold usage as well as inconsistency in the distance between and type of adhesion posts complicates initial establishment of the technique with confidence. We describe an inexpensive, but readily adaptable silicone chamber system for the generation of skeletal muscle constructs that can readily be standardized and used to elucidate myoblast behavior in a three-dimensional space. Muscle generation, regeneration and adaptation can also be investigated in this model, which is more advanced than differentiated myotubes.

## Introduction

Three-dimensional (3D) skeletal muscle constructs can be bioengineered *in vitro*. 3D models have advantages over 2D cell cultures in mimicking *in vivo* conditions as they allow for the study of dimensionality, cellular architecture, cell polarity and function. Constructs can be adapted for the generation of *in vitro* drug screening assays as well as *in vivo* tissue repair following transplantation of constructs (Vandenburgh et al., 2008; Corona et al., 2012). If genetically modified to express recombinant protein, these constructs can be used for therapeutic protein delivery (Vandenburgh et al., 1996).

An assortment of methods for the generation of bio-artificial skeletal muscle have been previously described (Table 1), with variations on aspects including chamber construction, matrix composition and ultimate tissue size generated. The chambers employed for 3D culture of skeletal muscle may be divided into two main categories: the uncomplicated silicone tubing model and the more intricate models constructed in chamber slides and multi-well plates (Table 1A) and chambers and micro-patterned wells that are precast via photolithographic moulds (Table 1B). Confluent myoblast monolayers cultured in a matrix-coated petri dish under differentiating conditions may also form scaffold-free 3D muscle tissue due to contractility of the differentiating fibers (Table 1C). These methods naturally reflect the thrust of the particular research group. While each model has specific advantages, key methodological aspects differ considerably between the various models which may potentially hamper efficient comparison. A critical overview of the various models is required before describing our simple chamber system.

**Table 1:** **A comparison of published methods for the generation of bio-artificial skeletal muscle**.

**Model description**	**Purpose**	**Anchor points**	**Cell type and seeding conditions**	**Matrix (final concentrations)**	**References**
**(A) CAST CHAMBERS CONTAINING TWO ADHESION POINTS**					
Silicone rubber tubes cut lengthwise in 35 mm dishes	Reversible gene therapy	Velcro pads or stainless steel mesh	C2C12 mouse myoblasts (1–4 × 10^6^ cells/well)	Collagen (1.6 mg/ml) and Matrigel (Ratio of 6:1 v/v)	Vandenburgh et al., 1996
Sylgard moulds produced in a vacuum-moulding process around Teflon spacers; 96-well plates	*In vitro* drug screening	Flexible silicone posts	Primary mouse myoblasts (0.2 × 10^6^ cells/well)	Collagen (1 mg/ml) and Matrigel (Ratio of 6:1 v/v) Fibrinogen–thrombin (0.5 mg/ml) and thrombin (1 U/ml)	Vandenburgh et al., 2008
Moulds cast from 2% agarose in PBS around Teflon spacers; 24-well plates	Heart muscle kinetics	Flexible Sylgard posts	Neonatal rat heart cells (0.62 × 10^6^ cells/well)	Fibrinogen (5 mg/ml) and Matrigel (100 μl/ml) polymerized with thrombin (32:1 v/v)	Hansen et al., 2010
Rectangular casting moulds (see Hansen et al., 2010)	Interaction between cells and surrounding matrix	Silicone pins	Primary human myoblasts (0.66 × 10^6^ cells/well)	Fibrin-based matrix	Chiron et al., 2012
Mechanical Cell Stimulator, version 4.0 (MCS4) Silicone rubber tissue moulds; 6-well plates	Mechanical stimulation to improve tissue-engineered human skeletal muscle	Stainless steel pins	Primary human skeletal muscle cells (1 × 10^6^ cells/well)	Collagen 1 (0.8 mg/ml) and Matrigel (Ratio of 6:1 v/v)	Powell et al., 2002
Silicone tube cut lengthwise (ends sealed with PDMS); 6-well plates	Hydrogel matrix combinations; influence on contractile function of engineered muscle tissue	Velcro adhesion pads	Primary rat skeletal myoblasts (6 × 10^6^ cells/well)	Collagen 1 (1.4 mg/ml) and Matrigel Fibrinogen (2, 4 or 6 mg/ml) and Matrigel (10%, 20% or 40% v/v)	Hinds et al., 2011
Commercially available single-well chamber slides	Optimized culture parameters improved reproducibility and the cellular architecture	Polyethylene mesh	Primary rat muscle derived cells (Cell count not stated; 3.2 ml/well)	Collagen 1	Smith et al., 2012
**(B) PHOTOLITHOGRAPHIC MOULDS AND MICRO-PATTERNED WELLS WITH POSTS**					
Sylgard tissue moulds cast from patterned master templates of coated photo-resistant silicone wafers	Muscle cell alignment	Array of silicone posts	C2C12 mouse myoblasts (1 × 10^6^ cells/well) Primary rat skeletal myoblasts (2 × 10^6^ cells/well)	Collagen I (1 mg/ml) and fibrinogen (2 mg/ml) (ratio of 1:0, 3:1, 1:1, 1:3, 0:1) Thrombin (0.4 U/mg fibrinogen) Matrigel added to all combinations	Bian and Bursac, 2009
Precast micro-patterned wells	Formation of muscle for use in bioactuators	PDMS cantilevers	C2C12 mouse myoblasts (400 cells/micro-patterned well)	Collagen 1 (2 mg/ml) and Matrigel	Sakar et al., 2012
**(C) SCAFFOLD-FREE CONFLUENT MONOLAYER CONTRACTION INTO CYLINDER BETWEEN PINNED SUTURES**					
Sylgard-based 35 mm culture dish coated with laminin	Excitability and contractile properties of muscle engineered from co-cultured primary cells	Silk sutures coated with 50 μg/ml laminin	Co-culture of primary myogenic precursors, fibroblasts and all related cell types	Laminin base layer (1 μ g/cm^2^)	Dennis and Kosnik, 2000
Sylgard-coated 35 mm culture dish coated with laminin	Skeletal muscle construct from C2C12 myoblasts; AIM-V media	Silk sutures	C2C12 myoblasts AIM-V serum-free medium; 0.02 × 10^6^ cells / dish	Laminin base layer (2 μg/ml) Laminin top layer (10 μg/ml)	Fujita et al., 2009b

The chambers employed for three-dimensional culture of skeletal muscle may be divided into three main categories: Cast chambers such as the uncomplicated silicon tubing model (A), Photolithographic moulds and micro-patterned wells (B), and Scaffold-free confluent monolayers (C).

These are fitted into the wells of standard culture plates.

The combination of hydrogel components as well as cell type, number and volume seeded per well vary considerably between published methods, and are summarized. The concentration of Matrigel is 10% (v/v) unless otherwise indicated.

In general culture vessels for 3D skeletal muscle constructs consist of tubes, standard pre-fabricated laboratory-based culture plates or dishes that contain two tissue adhesion points that mimic tendons and consist of either cast silicone posts, metal pins and mesh, sutures or Velcro pads (Vandenburgh et al., 1996, 2008; Dennis and Kosnik, 2000; Powell et al., 2002). Some models require post-modification with custom-made inserts that replace pins or Velcro adhesion points. In the models consisting of a silicone tube or a precast chamber slide, the adhesion points are on average 18–30 mm apart, which may dictate factors including the volume and concentration of cells initially seeded (Table 1A) (Powell et al., 2002; Vandenburgh et al., 2008). The cell seeding number ranged from 1 × 10^6^ to 6 × 10^6^ cells per tube, and the hydrogel-cell suspension volume varied between 400 μ l required for the tube models and 3.2 ml for the adapted single well-chamber slide (Table 1A) (Vandenburgh et al., 1996; Hinds et al., 2011; Smith et al., 2012). The matrix mixture and culture periods are similarly diverse. This is, however, also an indication of the ease with which tissue-engineering models that use silicone tubes with adhesion points may be constructed and adapted for a range of purposes (Vandenburgh et al., 2008; Hinds et al., 2011; Smith et al., 2012).

While Vandenburgh initially generated *in vitro* 3D muscle tissue constructs from C2C12 myoblasts in silicone tubing containing Velcro pads ~25 mm apart (Table 1A), this group subsequently employed a more complex custom-built silicone mould cast around a Teflon template with two flexible silicone posts inserted into a standard 96-well plate 4 mm apart (Vandenburgh et al., 1996, 2008). In other more recently-developed models these anchorage points and the distance between them in custom-built models varied from 4 to 50 mm, according to the size of the wells and aspects required for the experiment. The flexibility of the cantilever posts in the more advanced models is an improvement from the originally employed metal pins or large, fixed Velcro pads or metal mesh (Vandenburgh et al., 2008). The advantage of these custom-built systems with standardized dimensions and with flexible cantilever posts is that the cultured constructs could be used to investigate muscle kinetics (force generation, internal strain or contractile forces) on mechanical stimulation, or in response to compounds such as insulin-like growth factor 1 and cholesterol-lowering statins (Eastwood et al., 1998; Nirmalanandhan et al., 2007; Vandenburgh et al., 2008; Smith et al., 2012).

The actual vessels used for myooid culture may also be custom-built via micro-pattern technology or photolithography (Table 1B) (Dennis and Kosnik, 2000; Vandenburgh et al., 2008; Fujita et al., 2009a; Hansen et al., 2010; Smith et al., 2012). Using this technology a negative template is generated and a mould is subsequently cast from biological grade silicone (Bian and Bursac, 2009; Sakar et al., 2012). The use of an array of posts in a wafer pattern rather than simply two adhesion points permits the formation of a sheet-like culture that allows for the investigation of muscle cell alignment. This is in contrast to the 3D construct generated between two cantilevers.

Even though reproducibility is improved, these casting methods and inserts demand specialized equipment, which commonly translates into an increase in cost. In addition the system may not easily be adaptable to different well-types or tissue sizes.

Currently, various hydrogel components are routinely used, both individually and in combination, to successfully engineer muscle tissue containing striated and aligned myotubes. These include collagen 1, Matrigel from Engelbrecht-Holm-Swarm (EHS) sarcoma cells, laminin I, and fibrin-based gels (Table 1) (Vandenburgh et al., 2008; Bian and Bursac, 2009; Hansen et al., 2010). The hydrogels differ in their molecular composition, macromolecular orientation and the degree of cross-linking (Dennis and Kosnik, 2000; Hinds et al., 2011). This has practical implications; for instance, Matrigel polymerization is temperature-sensitive, while laminin I and collagen 1 are able to spontaneously form 3D gels at room temperature (Yurchenco et al., 1992). Furthermore, myogenesis itself is differentially affected by these matrix factors; collagen 1 has been shown to suppress differentiation, whereas Matrigel promotes myotube formation in both 2D and 3D models (Langen et al., 2003; Grefte et al., 2012). Laminin I promotes myoblast adherence, proliferation and myotube fusion (Schuler and Sorokin, 1995; Vachon et al., 1996). Finally, the matrix combinations as reported for the different models vary considerably (Table 1). The most conventional combination consists of collagen 1 in combination with Matrigel in a ratio of 6:1 (v/v) or a 10–20% Matrigel component (Table 1). The concentration of fibrinogen on its own or used in combination with other matrix components also varies considerably (Table 1) (Vandenburgh et al., 2008; Hinds et al., 2011). Such diversity may hamper direct comparison of results.

Successful formation of scaffold-free cylindrical 3D myooids in 35 mm culture dishes coated first with Sylgard and then laminin has also been described (Dennis and Kosnik, 2000; Fujita et al., 2009b) (Table 1C). After an initial low seeding density (2–10 × 10^3^ cells) in a coated 35 mm dish, cells are cultured in a 2D monolayer to confluence. This is followed by replacement of the growth media (GM) with differentiation media (DM) to stimulate differentiation into muscle fibers. Subsequent formation of cylindrical myooids is due to contraction of the fibers and release from the underlying Sylgard coating (Huang et al., 2005; Fujita et al., 2009a). Silk sutures pinned into the Sylgard base act as handling points and mimic flexible tendons for the cylindrical myooid. The laminin matrix employed merely forms a separating layer between the Sylgard coating and the cultured cells, while the final histology and molecular characteristics reflect that of skeletal muscle. It is believed that the interaction between co-cultured primary fibroblasts and myoblasts in this model allows for the investigation of functional and molecular development, as reflected by the contractile properties and expression of transcription factors MyoD and muscle-related myosin heavy chain of such fabricated skeletal muscle (Dennis and Kosnik, 2000; Huang et al., 2005; Fujita et al., 2009b). The culture period to establish this model is, however, considerably longer than the previously-mentioned models.

Although numerous methods for bioengineering skeletal muscle exist, currently no standardized culture vessel and protocol has been proposed. Also, many are prohibitive due to the required customized equipment and intricate casting techniques. Below we describe an inexpensive, accessible hydrogel-based system that may be readily standardized, yet is easily customized to reflect desired matrix combinations and tissue size. This model is ideal for laboratories expanding into three-dimensional assays.

## Materials and methods

### Selection and construction of chamber

**Figure 1:**
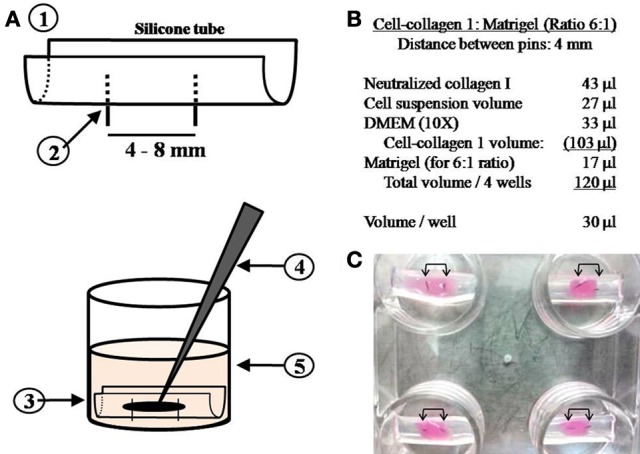
**Details on simple silicone tube chamber construction and hydrogel-cell preparation. (A)** Biological grade silicone tubing is cut to fit the diameter of the well; the tube is then cut lengthwise in half (1). Surgical grade stainless steel pins are inserted through the silicone wall at predefined distances from each other (2). The silicone tube is secured in place within the well with Sylgard 182 to form a chamber (3). The hydrogel-cell suspension is pipetted into the silicone chamber around the pins (4) and the well is flooded with growth media once the gel construct has set (5). **(B)** Calculations for preparation of the hydrogel-cell mixture (Ratio of 6:1). **(C)** A multi-well plate containing a hydrogel-C2C12 mix seeded in silicone tube chambers (arrows indicate pins positioned 4 mm apart).

An adaptable chamber system was generated by using 18 mm sections of biological grade silicone tubing (outer diameter: 5 mm) which was cut in half (lengthwise) (Figure 1A). Surgical grade stainless steel pins (3 mm long, outer diameter: 0.2 mm) were inserted into the tubes to act as adhesion points; distances between the pins ranged from 4 to 8 mm, depending on the size of the muscle tissue required. The tubes were fitted into each well of a 4- or 24-well-culture plate and secured within the wells using Sylgard 182 (Dow Corning Corporation, cat. 3097358-1004) (Figure 1A). Sylgard was allowed to cure for 24 h and the plates sterilized overnight under an ultraviolet light.

### Selection and preparation of cells

Murine C2C12 myoblasts (ATCC, cat. CRL-1772) were maintained in GM containing Dulbecco's Modified Eagle Serum (DMEM, Highveld, cat.CN3193-9), L-glutamine (2% v/v, Cambrex, cat.17-605E), PenStrep (2% v/v, Cambrex, cat.17-602E), and Fetal Calf Serum (FCS; 10% v/v, Invitrogen, cat.10108165). Primary cultured human skeletal myoblasts (HSKM, Lonza, cat. CC-2561) were cultured in Ham's-F10 (Gibco, cat.15140), FCS (20% v/v), Penstrep (2% v/v), L-glutamine (2% v/v), fibroblast growth factor (FGF; 2.5 ng/ml, Promega, cat.G507A).

### Preparation of hydrogel/matrix

Rat tail collagen 1 (3.6 mg/ml; Sigma, cat. C9791) was neutralized with 10% NaOH (~30 μl per ml of collagen 1) until a color change was observed (yellow to pink due to pH indicator in DMEM). All solutions were kept on ice to restrict matrix polymerization prior to seeding.

### Cell-hydrogel suspension

Initially a cell suspension (27 μl; in GM) containing 3.2 × 10^6^ HSKM cells or 6.4 × 10^6^ C2C12 cells and 33 μl 10X DMEM was added to the neutralized collagen 1 solution (43 μl) (total volume: 103 μl). To achieve a cell-collagen 1:Matrigel ratio of 6:1 (v/v) as is often used in the generation of skeletal muscle (Vandenburgh et al., 1996, 2008; Powell et al., 2002), 17 μl Matrigel (10.1 mg/ml stock concentration, BD Biosciences, cat.356231) was added to the cell-collagen 1 suspension to achieve a final volume of 120 μl (Figure 1B).

HSKM cells are larger than C2C12 myoblasts; this accounts for the lower number of human myoblasts in the hydrogel mix when compared to mouse myoblasts. The cell/hydrogel suspension containing C2C12 or HSKM cells was pipetted into each silicone tube chamber around the pins. Constructs were subsequently incubated at 37°C overnight and the wells then flooded with 350 μl GM (Figure 1A). Twenty-four hours later, culture GM was replaced with DM, which contained DMEM, L-glutamine (Lonza; 2% v/v), PenStrep (Lonza; 2% v/v) and horse serum (HS; 1% v/v, Invitrogen, cat.16050-130).

### Preparation for image collection

Brightfield images were captured at various stages of muscle development using a Motic 3.0 MP camera and an Olympus stereo microscope (VMZ, Japan). For histochemical investigation of desmin expression and the actin cytoskeleton, muscle constructs were fixed for 2 h in the wells in paraformaldehyde (4% prepared in PBS). Subsequent removal of the pins allowed constructs to be transferred from the chambers to culture wells where they were incubated with either a polyclonal rabbit anti-desmin antibody (1/600, Abcam cat.AB15200) for 2 h at room temperature followed by a Dylight488 donkey anti-rabbit antibody (1/1000, Jackson, cat.711-485-152) for 1 h at room temperature, or TRITC-conjugated Phalloidin (0.5 ng/ml, Sigma; cat. P1951) for 2 h at room temperature. Nuclei were stained with Hoechst (2.5 μg/ml, Sigma, cat C8890) for 10 min at room temperature and constructs mounted on glass slides with Moviol and viewed with the Zeiss 710 confocal microscope. Constructs cultured for 15 days in DM were also fixed with glutaraldehyde (2%, 2 h at room temperature), dehydrated with a graded series of alcohols and embedded in Spurr's resin. Thin sections were cut and DIC images were obtained with the 710 Zeiss confocal microscope.

**Figure 2:**
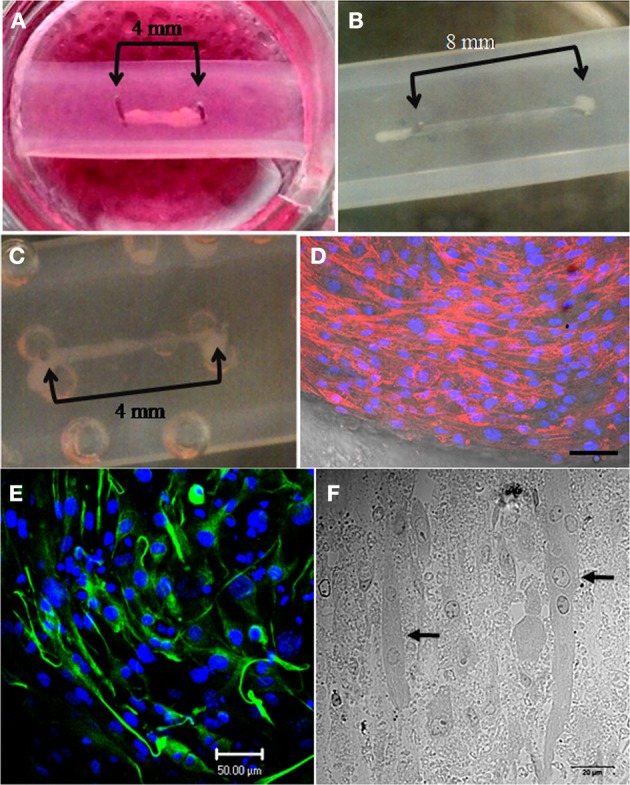
**Successful generation of mouse and human skeletal muscle constructs using the simple silicone chamber system. (A)** When seeded in a matrix of collagen 1 and Matrigel (14%), mouse C2C12 cells formed tissue (day 7 in differentiation media) between pins placed 4 mm apart. **(B)** When seeded in a matrix of collagen 1 and Matrigel (14%), mouse C2C12 cells formed tissue (day 3 in differentiation media) between pins placed 8 mm apart. **(C)** When seeded in a matrix of collagen 1 and Matrigel (14%), human skeletal muscle (HSKM) cells formed tissue (day 3 in differentiation media) between pins placed 4 mm apart. **(D)** After 12 days in differentiation media, actin fibers stained with TRITC-phalloidin and were clearly visible in the differentiated mouse C2C12 myotubes. Nuclei were stained with Hoechst (scale bar = 50 μm). **(E)** After 15 days in differentiation media, elongated myotubes were aligned and contained desmin, an intermediate filament required for myotube contractile function (scale bar = 50 μm). **(F)** Thin sections of resin-embedded C2C12 myoblasts culture for 15 days in differentiation media showed the formation of multi-nucleated (arrows) myotubes (scale bar = 20 μm).

This adapted technique proved to be advantageous for skeletal muscle formation with constructs from both C2C12 and HSKM cells successfully spanning the pins after a 3–7 day culture period (Figures 1C, 2A–C). After 12–15 days in culture, differentiated C2C12 myotubes showed clear formation of actin fibers (Figure 2D). In addition, aligned myotubes expressed desmin (Figure 2E) and longitudinal sections showed evidence of organization into multinucleated myotubes (Figure 2F), which is an initial requirement for functionality.

## Discussion

Muscle tissue engineering is no longer in its infancy, nor is it the prerogative of only a few laboratories. Nonetheless, only a few research groups have made major advances in this field (Vandenburgh et al., 2008; Hansen et al., 2010; Vandenburgh, 2010; Chiron et al., 2012; Corona et al., 2012; Sakar et al., 2012; Smith et al., 2012). One of the major hurdles when initially trying to establish *in vitro* tissue engineered muscle constructs is the lack of consistency across published methodology. Such lack of uniformity was highlighted by a summary of the range of moulds already employed in muscle tissue engineering (Table 1). A careful view of Table 1 indicates that particular moulds have been designed with specific purposes in mind (Powell et al., 2002; Bian and Bursac, 2009; Sakar et al., 2012). However, differences in cell type, seeding density as well as variability in hydrogel/scaffolds used, and also variations in the distance between and type of adhesion posts (i.e., stainless steel, sutures, Velcro, etc.) hampers initial establishment of the technique with confidence. In the current study we describe an inexpensive, readily adaptable silicone chamber system for the generation of skeletal muscle constructs.

We highlight the basic steps and requirements needed to form differentiated muscle tissue from either C2C12 or HSKM myoblasts in a collagen 1/Matrigel hydrogel. This model is adaptable to fit into any existing culture dish or chamber used in most laboratories. Despite this simplicity, variations in the distance between pins, as well as cell number and matrix-cell volume can be readily achieved. In addition, the use of appropriate pins as anchor points allows for future mechanical stimulation to investigate contractile forces and allows for the study of internal stresses during muscle differentiation in 3D cultures. It is also useful for investigations into genetic manipulation, drug therapy or co-culture of complimentary cell phenotypes.

This model has several practical advantages. Biological grade silicone is inexpensive, readily available and allows for ease of pin insertion and subsequent tissue manipulation. In addition, after initial use, the various components of the chamber system may also be reused following de-cellularization with ammonium hydroxide and cleaning with alcohol and sonication. This model, with standardized parameters, may be used as an optimized system for initial evaluation of factors involved in skeletal muscle generation from both primary cultured myoblasts and established cell lines.

Imaging of myoblasts functioning in a three-dimensional space is also more closely aligned to *in vivo* behavior. With this model we have described the expression of desmin, an intermediate filament that plays a key role in the integration of striated muscle morphology and function (Capetanaki et al., 2007). We also showed the transition of myoblasts into elongated, multi-nucleated myotubes, one of the relevant steps during myoblast differentiation. It must be noted, however, that the use of C2C12 myoblasts to generate functional striated tissue may require electrical stimulation during the generation of the bioengineered tissue; this is not the case for primary muscle progenitor cells (Langelaan et al., 2011). Engineered tissue may further be processed for histological purposes or immunocytochemical investigation of transcription factors and expressed proteins. We propose that the method we describe may allow skeletal muscle research groups utilizing 2D cell culture models to move into 3D tissue models with relative ease. This will be important to enable more rapid enhancement of our understanding of muscle synthesis, repair and adaptation *in vitro*, in a model more advanced than differentiated myotubes.

### Conflict of interest statement

The authors declare that the research was conducted in the absence of any commercial or financial relationships that could be construed as a potential conflict of interest.
